# Burden of disease due to dementia in the elderly population of Korea: present and future

**DOI:** 10.1186/1471-2458-13-293

**Published:** 2013-04-03

**Authors:** Jae-Hyun Park, Jin-Hee Eum, Bolor Bold, Hae-Kwan Cheong

**Affiliations:** 1 Department of Social and Preventive Medicine, Sungkyunkwan University School of Medicine, 2066 Seobu-ro, Jangan-gu, Suwon, Gyeonggi-do, 440-746, Republic of Korea; 2 Center for Molecular Medicine, Samsung Biomedical Research Institute, Seoul, Republic of Korea

**Keywords:** Dementia, Burden of disease, DALY, Alzheimer's disease

## Abstract

**Background:**

With the rapid aging of populations around the world, dementia has become one of the most important public health problems in Eastern Asian countries. The purpose of the present study was to provide an estimate of the burden of dementia and forecast its future burden, as generalized to the Korean population, and to provide detailed gender- and age-specific information regarding the burden of dementia in the elderly population of Korea.

**Methods:**

‘Disability-adjusted life years’ (DALYs) were used to estimate the burden of dementia. Epidemiologic data from national statistics and nationwide epidemiologic studies in the year 2008 were used to obtain representative outcomes for the Korean population. We estimated the DALYs due to dementia from the years 2010 to 2050 by applying demographic structure projections in terms of 5-year age groups in Korea.

**Results:**

The burden of disease due to dementia in Korea is 528 per 100,000 population (males: 435, females: 622) and 5,117 per 100,000 in those over the age of 65 years (males: 5,228; females: 5,041); this accounts for 4.5% of the total burden of disease in the year 2008. In the year 2050, DALYs due to dementia (814,629) are expected to be 3.0 times higher than those in the year 2010 (274,849).

**Conclusion:**

Dementia has the highest burden of disease in the elderly Korean population, and this burden will increase sharply with the aging of the population. More comprehensive and multi-dimensional approaches, including clinical, psychological, social, and political means will be needed for the management of the dramatically increasing burden of dementia.

## Background

Dementia generally occurs in the elderly and is one of the most serious mental illnesses in the Korean population [1]. Given its progressive, irreversible natural course, together with its high prevalence in the elderly population, dementia has one of the biggest burdens of disease in the elderly [2,3]. As the world’s population is rapidly aging, dementia has become an important public health problem [4,5]. The World Health Organization (WHO) has estimated dementia to be the 11th leading cause of years lived with disability (YLD) at a global level, accounting for 2.0% of the total global YLDs [6]. By the year 2050, the worldwide prevalence of dementia will quadruple to 106.2 million persons, with 1 in 85 persons living with Alzheimer's disease [7]. Specifically, dementia is expected to become a more of a serious health and social burden in developing countries than in developed countries. This is because unprecedented declines in mortality and fertility have resulted in a rapid aging process in most developing countries [8], which is exacerbated by poor health care facilities.

To estimate the burden of diseases, the WHO recommends using disability-adjusted life years (DALYs) [9]. DALYs have the merit of simplicity and comprehensiveness, as its calculation produces a single number that combines information on mortality and non-fatal health outcomes. According to the Global Burden of Disease (GBD) estimates from the 2003 World Health Report [10], dementia contributed 11.2% of the YLD in people aged 60 years and older. This is more than stroke (9.5%), musculoskeletal disorders (8.9%), cardiovascular disease (5.0%), and all forms of cancer (2.4%).

These estimates have limitations because of the lack of accurate epidemiologic data, especially in developing countries [7]. In 2003, the World Health Report addressed dementia in many world regions; however, such evidence suffers from incompleteness or is limited in coverage. Even when a wider evidence base is available for country- or region-specific figures, these estimates are sometimes generated from a single study with limited generalization. Previous global estimates of the number of people with dementia have tended to apply a uniform age-specific prevalence, assuming no geographic variation [11,12]. However, prevalence has been noted to be lower in developing countries [13], strikingly so in some studies [14,15].

Korea represents an unique situation in terms of the burden of dementia. Korea has a population of over 49 million people. Its life expectancy at birth has increased dramatically in recent years (71.3 in 1990, 78.6 in 2000, and 80.8 in 2010 [16]. The country is in the midst of a rapid transition from being a low-income country to becoming a high-income country, and its population structure is aging at an unprecedented speed [16]. The proportion of the elderly population reached 7.2% in 2000 and is expected to exceed 14% by 2018, and 20.8% by 2026; one of the fastest transitions from an aging society to an ultra-aged society. Therefore, changes in the burden of disease due to dementia in Korea may represent a typical situation that will be experienced in many developing countries in the near future.

A previous WHO study [17] estimated the burden of dementia in Korea using several epidemiologic indices adopted from other countries. These indices included incidence and relative risk of mortality from European countries, where prevalence was consistent with literature for Chinese populations due to the lack of epidemiologic data from the Korean population. However, a recent study [18] also did not use epidemiologic data produced in the Korean population, but used meta-analysis results from other high-income Asia Pacific countries instead. As such, there is a critical need for a study to estimate the burden of dementia using Korean-specific epidemiologic data.

This study was conducted to provide an estimate of the burden of dementia in the elderly and to forecast it for the future to provide detailed gender- and age-specific estimates of the burden of dementia using the most recent epidemiologic data from the Korean population.

## Methods

### Data source and epidemiologic data

DALYs due to dementia were calculated using various epidemiologic data such as incidence, prevalence, remission rate, case mortality, age of onset, disease duration, disability weight, total mortality of the population, and population structure. All the data were derived from various epidemiologic studies conducted in Korea and from a Korean national dataset [19]. This previous nationwide population study carried out in 2008 was based on 8,199 probability-sampled Koreans aged 65 years or older. The study used a 2-step assessment method to identify persons with dementia: 1) All the sampled individuals were invited to participate in the Phase I screening assessment using the Mini-Mental State Examination via door-to-door home visits, and 2) the Phase II diagnostic assessment for dementia was administered using the Korean version of the Consortium to Establish a Registry for Alzheimer’s Disease Assessment Packet (CERAD-K) clinical assessment battery, neuroimaging, including computed tomography or magnetic resonance imaging (MRI), and laboratory testing. Patients were diagnosed with dementia using the criteria established in the Diagnostic and Statistical Manual, 4th Revision (DSM-IV), or with Alzheimer’s disease according to the criteria of the National Institute of Neurological and Communicative Disorders and Stroke and the Alzheimer’s Disease and Related Disorders Association (NINCDS-ADRDA), or with vascular dementia according to the criteria of the National Institute of Neurologic Disorders and Stroke/Association Internationale pour la Recherche et l’Enseignement en Neurosciences (NINDS-AIREN) [19]. We adopted gender- and age-specific prevalence of dementia for this study from the published article [19] and report [20]. As the previous report used a sample aged 65 years or older, we also adopted the prevalence of dementia in adults aged 65 years or older. Case mortality due to dementia and total mortality of the population by gender and age group were derived from cause of death statistics from Statistics Korea [21]. In Korea, the cause of death on the death certificate must, by law, be decided by a doctor. Therefore, Korea has a relatively comprehensive and accurate death certification system. The population structure of each gender and age group in 2008 was derived from baseline population estimates from Statistics Korea [16]. These datasets from Statistics Korea are public data sources and completely open to the public with free website access [16,21]. Other parameters for the estimation of the burden of disease, including incidence, disease duration, and age of onset, were estimated with DISMOD-II software [22], using prevalence, remission rate, case mortality, total population, and total mortality. DISMOD II, developed by WHO for the GBD project, is a software program that can be used to estimate missing parameters and to verify the consistency of the estimates for diseases [23]. The remission rate was assumed to be zero. The disability weight of 0.911 was derived from the results in the previous Korean panel study [24].

### DALY calculation and projection

In general, this study followed the protocols of the original GBD study [25]. DALYs are the sum of two components. The first, Years of Life Lost (YLLs), measures the number of years lost when a person dies prematurely. The second component, Years Lived with Disability measures the number of years of healthy life lost due to temporary or permanent disability. YLL estimates were derived from mortality and life expectancy at birth in 2008 [26]. YLD estimates were derived from incidence, mortality, disease duration, and age of onset from the DISMOD II output. YLL, YLD and DALY estimates were calculated according to gender and 5-year age groups. We forecasted the DALYs due to dementia in the years 2010–2050 by applying demographic structure projections and assuming a constant age-specific prevalence of dementia [27].

## Results

### Results of DISMOD II modeling

Input variables in the DISMOD II model (prevalence, dementia mortality, remission rate, population and overall mortality) and output variables in the year 2008 generated from the model are illustrated in Table 1. Comparing input and output variables in terms of prevalence we did not find any discrepancies; this implies consistency of estimates for the diseases. Incidence demonstrated its peak at the ages of 80–84 and subsequently decreased. Case mortality showed a gradually increasing pattern in both genders. Incidence, prevalence, mortality, duration, and age of onset outputs shown in Table 1 were used in the YLD calculation. Mortality due to dementia from the DISMOD II output was used in the YLL calculation.

**Table 1:** Input and Output Variables in the DISMOD II Model (year 2008)

**Age group**	**Input**								**Output**									
	**Prevalence**^**‡**^		Dementia mortality ^*^		Population^**^		Mortality^*^		**Incidence**		**Prevalence**		**Mortality**		**Duration (yr)**		**Age of onset (yr)**	
	**Men**	**Women**	**Men**	**Women**	**Men**	**Women**	**Men**	**Women**	**Men**	**Women**	**Men**	**Women**	**Men**	**Women**	**Men**	**Women**	**Men**	**Women**
65–69	0.0352	0.0307	0.0001	0.0001	829,526	987,096	0.0199	0.0077	0.0077	0.0062	0.0194	0.0171	0.0010	0.0003	12.61	16.69	67.85	67.85
70–74	0.0571	0.0436	0.0004	0.0003	610,634	825,685	0.0341	0.0150	0.0099	0.0078	0.0609	0.0517	0.0005	0.0003	10.99	13.73	72.45	72.44
75–79	0.1204	0.1152	0.0009	0.0010	345,459	590,729	0.0564	0.0298	0.0177	0.0135	0.1146	0.0936	0.0008	0.0008	8.20	10.06	77.85	77.86
80–84	0.2163	0.1628	0.0029	0.0031	163,459	345,906	0.0968	0.0585	0.0280	0.0278	0.2098	0.1759	0.0023	0.0025	6.32	7.46	82.43	82.64
85–89	0.2698	0.2871	0.0070	0.0077	65,151	167,598	0.1592	0.1087	0.0172	0.0303	0.2850	0.2787	0.0058	0.0067	5.29	5.73	87.13	87.31
90+	0.2698	0.2871	0.0128	0.0153	17,951	66,832	0.2437	0.1992	0.0080	0.0108	0.2818	0.3064	0.0118	0.0143	4.53	3.87	99.71	97.06

‡Kim KW et al. [19], *Statistics Korea [21], **Statistics Korea [16].

### YLLs, YLDs, and DALYs due to dementia

**Figure 1:**
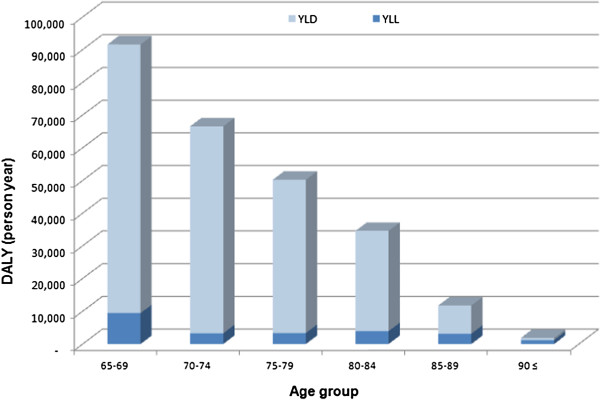
**YLL, YLD, and DALY due to dementia in Korea (2008).** YLL: years of life lost, YLD: years lived with disability, DALY: disability-adjusted life years.

**Figure 2:**
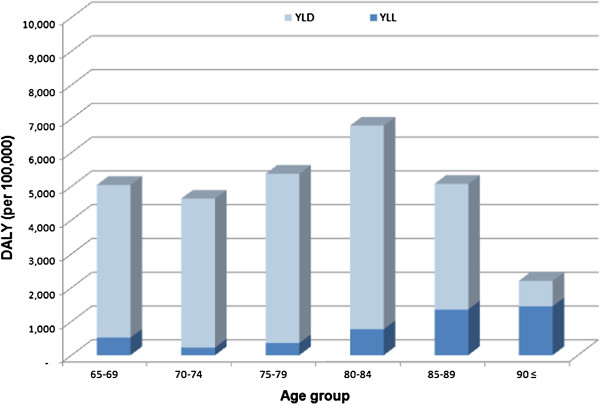
**YLL, YLD, and DALY due to dementia in Korea (2008) (per 100,000 in each age group).** YLL: years of life lost, YLD: years lived with disability, DALY: disability-adjusted life years.

YLLs, YLDs, and DALYs in the year 2008 due to dementia in the elderly in Korea were 24,344, 232,303, and 256,647 person-years, respectively (Table 2). The burden of dementia was highest in the age group of 65–69 years (Table 2, Figure 1). YLLs, YLDs, and DALYs due to dementia were calculated per 100,000 in each age group and are listed in Table 2 and Figure 2. In total, the YLLs, YLDs, and DALYs in 2008 due to dementia per 100,000 were 50, 478, and 528 person-years, respectively. When projected to the general population, the burden of disease due to dementia per 100,000 was 435 person-years in males and 622 person-years in females in 2008. In the population aged 65 years or older, the burden of dementia was 5,117 person-years per 100,000 (males: 5,228 person-years, females: 5,041 person-years) in 2008.

**Table 2:** YLL, YLD, and DALY due to Dementia in Korea (year 2008)

**Age group**	**Men**			**Women**			**Total**			**Men (per 100,000 people)**			**Women (per 100,000 people)**			**Total (per 100,000 people)**		
	**YLL**	**YLD**	**DALY**	**YLL**	**YLD**	**DALY**	**YLL**	**YLD**	**DALY**	**YLL**	**YLD**	**DALY**	**YLL**	**YLD**	**DALY**	**YLL**	**YLD**	**DALY**
65–69	6,377	38,325	44,703	3,065	43,817	46,882	9,442	82,142	91,585	655	3,934	5,389	311	4,439	4,749	520	4,522	5,041
70–74	1,663	28,061	29,725	1,595	35,221	36,815	3,258	63,282	66,540	201	3,383	4,868	193	4,266	4,459	227	4,406	4,633
75–79	1,152	18,328	19,480	2,197	28,563	30,761	3,349	46,892	50,241	189	3,002	5,639	372	4,835	5,207	358	5,009	5,367
80–84	1,039	8,823	9,862	2,888	21,870	24,757	3,927	30,693	34,620	301	2,554	6,034	835	6,322	7,157	771	6,026	6,797
85–89	684	1,477	2,161	2,457	7,179	9,636	3,141	8,656	11,797	418	904	3,317	1,466	4,283	5,750	1,350	3,719	5,069
90+	201	114	315	1,026	524	1,549	1,227	638	1,864	308	175	1,754	1,535	784	2,318	1,447	752	2,199
Total	11,116	95,130	106,246	13,228	137,174	150,401	24,344	232,303	256,647	46	390	435	55	567	622	50	478	528
65 ≤	11,116	95,130	106,246	13,228	137,174	150,401	24,344	232,303	256,647	547	4,681	5,228	443	4,597	5,041	485	4,631	5,117

YLL: years of life lost, YLD: years lived with disability, DALY: disability-adjusted life years.

### DALYs due to dementia projected from the years 2010–2050

**Figure 3:**
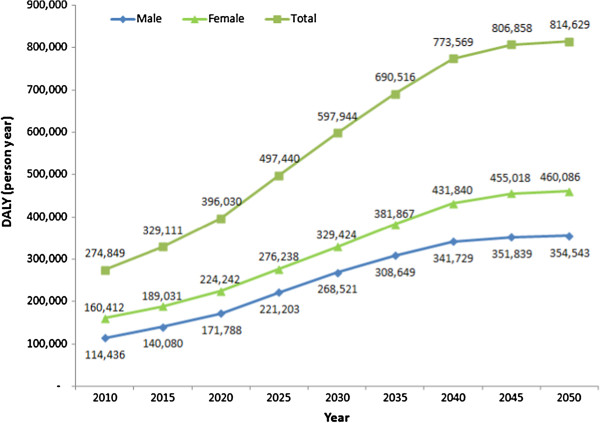
**DALYs due to dementia projected from the years 2010 to 2050 in Korea.** DALY: disability-adjusted life years.

DALYs due to dementia projected from the years 2010 to 2050 are illustrated in Figure 3. In both males and females, DALYs due to dementia were expected to show a continuous increase. In 2050, DALYs due to dementia (814,629 person-years) are expected to be 3.0 times higher than those in the year 2010 (274,849 person-years).

## Discussion

In this study, we estimated the burden of disease due to dementia in Korea as 528 per 100,000, which accounts for 4.5% of the total burden of disease in Korea in the year 2008. WHO previously estimated the burden of disease due to dementia in Korea as 222 per 100,000 in the year 2004 [28]. This accounted for 1.7% of the total burden of disease in Korea which is less than half of the result reported in this study. This gap is derived from differences in the epidemiologic data used in the studies. The WHO study estimated the burden of dementia in Korea based on the fact that Korea belongs to the Western Pacific Regional Office (WPRO) B1, which includes China [17]. The WHO study also used overall weighted prevalence of dementia of 3.54% (male) and 4.45% (female) in calculating the DALYs in the WPRO B1 region. However, the prevalence of dementia used in this study was around 8.1% in 2008, which is also higher than the results of another recent study in which the standardized prevalence for adults aged 60 years and over is 6.3%) [18]. As addressed in the methods section, the prevalence of dementia used in this study was derived from the most recent study using a representative nationwide sample of elderly Koreans [19]. In addition, Korea is one of the most aged societies in the WPRO B1 region and shows the most rapid rate of aging among developing countries [29]. Previous studies have shown that the prevalence of dementia among the elderly ranges from 3.6–11.9% in Western countries [30-36] and 4.8–7.2% in Japan [37-40], which is a similar or lower prevalence compared to that of Korea. These facts could explain Korea’s high burden of dementia.

This study and the previous WHO study also differed in their disability weight of dementia used in the DALY calculation. In this study, we used the disability weight of 0.911, which was 1.46 times higher than the disability weight of 0.625 used in the WHO study [17]. Whereas disability weights in the WHO study were greatly influenced by developed Western countries, the disability weights in this study were derived from a study conducted in Korea, reflecting psychosocial circumstances specific to the country of study [24]. This gap, in part, explains the higher DALY in this study compared with the WHO study.

Regarding the incidence and prevalence rates of dementia used in the DALY calculation in this study, increasing trends with increasing age in this study correspond with previous study results. Several previous studies [7,41,42] have reported that the doubling time of incidence and prevalence of dementia was approximately 5 years. In this study, the incidence and prevalence of dementia increased to almost double as age increased by 5 years, until the ages of 85–89 years. Above the age of 90 years, the rates of increase slowed and subsequently decreased. In a previous study [8], the increasing occurrence of dementia appeared to curtail at age 85 and reached a plateau in people aged 85 years and older. This finding may relate to the difficulty in studying samples of individuals aged 90 years and older; the degree of diagnostic difficulty increases with age. These findings resulted in the increasing trend of DALY up to the age of 80 years followed by a decrease in DALY. However, even though we could not obtain an accurate level of incidence or prevalence of dementia in people aged 80–90 years and older, the population in that age group is relatively small; therefore a more accurate estimation of DALYs due to dementia may not significantly influence the overall figure.

The 274,849 DALYs due to dementia in 2010 will rise to 814,629 DALYs in 2050, a 3.0-fold increase. This increase could in part be explained by the rapidly aging population in Korea [29]. It also indicates that other developing countries shall face a tremendous increase in the burden due to dementia in the near future. Previous reports have also forecasted that the burden of dementia will become more serious in developing countries than in developed countries because unprecedented declines in mortality and fertility have resulted in a rapidly aging population in most developing countries [8]. This forecast provides us with important insight regarding health and welfare service systems in developing countries. Previously, most developing countries have focused on communicable diseases and some chronic diseases as the major health problems. These countries should now prepare to manage diseases like dementia, which hamper the quality of life and autonomy of older people. This will require the redesign of national health and welfare service systems in developing countries.

This study has several limitations. First, because of the lack of more detailed epidemiologic information, we could not distinguish the severity of dementia and, as such, could not estimate DALYs according to disease severity level. The resources needed to care for dementia patients depend on the stage of the disease. Adult daycare programs may be adequate in the early stages, while a high level of care, equivalent to that in nursing homes, will be needed in the late stages [7]. Second, we could not distinguish the type of dementia, i.e., vascular dementia (VaD) *vs*. Alzheimer's disease (AD). Even though there is a small difference regarding the quality of life or autonomy due to the type of dementia, the risk factors are different in each type, meaning different strategies for prevention are necessary. According to previous studies, the ratio of VaD to AD has shown a wide variation, ranging from 1:1.5 – 1:7 according to the country. In Asian countries, the ratio of VaD to AD is around 1:1.5. Most recent studies in Korea have reported a ratio up to 1:3 [18,42], demonstrating that the epidemiology of dementia in Korea is approaching that of Western countries. These different types of dementia might manifest as different levels of DALYs. However, because VaD and AD share similar levels of deterioration in quality of life and autonomy, the different types of dementia should not impose substantial inaccuracies in the DALY calculation. Third, in projecting the burden of dementia, we assumed that the age-specific prevalence of dementia would remain constant over time. In fact, changes in risk exposure may increase or decrease the incidence. Improved medical and social care might reduce case mortality and increase prevalence. Interventions that delay onset would have substantial potential for reducing age-specific prevalence [7,29,43]. In light of these points, the future burden of dementia is expected to be different compared to that found in this study.

Despite these limitations, we believe that the detailed estimates contained in this paper provide the best currently available basis for policymaking, planning, and allocation of health and welfare resources for the management of dementia. To tackle these problems, continuous monitoring of the burden of disease due to dementia should be conducted from this point forward. Raising awareness and creating a framework for positive engagement between policymakers, clinicians, researchers, caregivers, and people with dementia will be needed in the near future.

## Conclusion

The burden of disease due to dementia in Korea is 528 per 100,000 population (males: 435, females: 622), and 5,117 per 100,000 in those over the age of 65 years (males: 5,228; females: 5,041). This accounts for 4.5% of the total burden of disease in the year 2008. In the year 2050, DALYs due to dementia (814,629) are expected to be 3.0 times higher than that in the year 2010 (274,849). This indicates that Korea shall face a tremendous increase in the burden due to dementia in the near future. This forecast provides us with important insight regarding health and welfare service systems in developing countries as well. Redesign of national health and welfare service systems in developing countries including Korea will be required in the near future.

## Abbreviations

AD: Alzheimer’s disease; CERAD-K: Korean version of the Consortium to Establish a Registry for Alzheimer's Disease Assessment Packet; DALY: Disability-adjusted life years; DSM-IV: Diagnostic and Statistical Manual, 4th Revision; GBD: Global burden of disease; MRI: Magnetic resonance imaging; NINCDS-ADRDA: National Institute of Neurological and Communicative Disorders and Stroke and the Alzheimer’s Disease and Related Disorders Association; NINDS-AIREN: National Institute of Neurologic Disorders and Stroke/Association Internationale pour la Recherche et l’Enseignement en Neurosciences; VaD: Vascular dementia; WHO: World Health Organization; WPRO: Western Pacific Regional Office of World Health Organization; YLD: Years lived with disability; YLL: Years of life lost

## Competing interests

The authors declare that they have no competing interests.

## Authors’ contributions

JHP prepared the manuscript and reviewed all the data and results. JHE and BB collected and analyzed the data, prepared tables and figures, and conducted reference work. HKC designed and participated in reviewing the data analysis, preparing the paper and finalizing the manuscript. All authors reviewed and were involved in the preparation and finalization of the manuscript. All authors read and approved the final manuscript.

## Pre-publication history

The pre-publication history for this paper can be accessed here:

http://www.biomedcentral.com/1471-2458/13/293/prepub
